# Aggressive and prosocial behaviors in adolescents from the department of Cordoba, Colombia

**DOI:** 10.1192/j.eurpsy.2024.1228

**Published:** 2024-08-27

**Authors:** E. P. Ruiz Gonzalez, M. N. Muñoz Argel

**Affiliations:** Universidad Pontificia Bolivariana, Montería, Colombia

## Abstract

**Introduction:**

Prosocial behaviors are voluntary behaviors that are performed for the benefit of other people and promote harmonious relationships with others. This type of enhanced behavior could reduce physical and verbal aggressive acts in adolescents.

**Objectives:**

analyze the association between aggressive and prosocial behaviors in adolescents

**Methods:**

The study was non-experimental of a transactional - correlational type, two evaluation instruments validated in the context were applied to 500 adolescents attending school in the department of Córdoba. The type of sampling was non-probabilistic.

**Results:**

A Pearson correlation was performed, previously verifying the normality of the data, which showed a statistically significant, negative association between the prosocial behaviors and the aggressive behaviors of those evaluated (Table 1).

**Table 1:** Correlation between prosocial behavior and aggressive behavior.

		PROSOCIAL BEHAVIOUR
**AGGRESSIVE** **BEHAVIOR**	Pearson correlation	-,197^**^
	Sig. (bilateral)	,004
	N	500

**Conclusions:**

Negative associations were identified between the two variables under study, that is, as prosocial behavior increases, aggressive behaviors could decrease. This finding serves as a basis for carrying out future intervention strategies in adolescents in the department of Córdoba.

**Disclosure of Interest:**

None Declared

